# Pregnancy Complications and Neonatal Mortality in a Serotonin Transporter Null Mouse Model: Insight Into the Use of Selective Serotonin Reuptake Inhibitor During Pregnancy

**DOI:** 10.3389/fmed.2022.848581

**Published:** 2022-03-10

**Authors:** Rafael R. Domingues, Milo C. Wiltbank, Laura L. Hernandez

**Affiliations:** ^1^Department of Animal and Dairy Sciences, University of Wisconsin-Madison, Madison, WI, United States; ^2^Endocrinology and Reproductive Physiology Program, University of Wisconsin-Madison, Madison, WI, United States

**Keywords:** pregnancy loss, prenatal mortality, perinatal mortality, genetic mouse model, selective serotonin reuptake inhibitor, sudden infant death, serotonin transporter

## Abstract

Selective serotonin reuptake inhibitors (SSRI) are widely prescribed to pregnant woman. Although some SSRI compounds are known to cause pregnancy loss and fetal malformations, other SSRI continue to be used by pregnant women. However, several studies have associated the use of SSRI with adverse pregnancy outcomes: intrauterine growth restriction, preterm birth, and neonatal morbidity. Nonetheless, interpretation of studies in humans are typically complicated by the adverse pregnancy outcomes caused by depression itself. Therefore, we used a mutant mouse model with genetic ablation of the serotonin transporter, the target site for SSRI, to unravel the role of the serotonin transporter on pregnancy outcomes. The serotonin transporter null mice had increased pregnancy loss (17.5 vs. 0%), decreased number of pups born (6.6 ± 0.2 vs. 7.5 ± 0.2), and increased neonatal mortality (2.3-fold). Furthermore, preterm birth, dystocia, and fetal malformations were only observed in serotonin transporter null mice. This genetically ablated serotonin transporter mouse recapitulates several adverse pregnancy outcomes similar to those in women undergoing SSRI treatment during gestation. Additionally, neonatal loss in the present study reproduced a sudden infant death phenotype as in humans and mice with altered serotonergic signaling. In conclusion, findings from this study demonstrate a role for serotonin transporter in pregnancy maintenance and neonatal health. Additionally, it suggests that the adverse pregnancy outcomes in women taking SSRI during gestation might be due to altered serotonin transporter function caused by SSRI independent of underlying depression. This is a critical finding, given the number of women prescribed SSRI during pregnancy, and provides the framework for critical research in this area.

## Introduction

Selective serotonin reuptake inhibitors (SSRI) are the primary class of antidepressants prescribed to pregnant women (1, 2). However, SSRI treatment during gestation has been associated with several adverse pregnancy outcomes: congenital malformations preterm birth, and increased neonatal morbidity (2–4). Moreover, some SSRI are not indicated for pregnant women (i.e., paroxetine) while others (sertraline, fluoxetine) are still widely used (5). Over the past decades, multiple studies have questioned the safety of SSRI for mother and infant (2, 4, 6–8).

Serotonin is a neurotransmitter and a hormone with a multitude of actions throughout the body ranging from embryo development to regulation of mood and behavior and regulation of vascular resistance (9, 10). A role for serotonin signaling in fetal neurodevelopment (11) and cardiac development (12, 13) has been established. Genetic ablation of serotonin receptors 2B (12) or 3A (14) results in embryonic and neonatal death due to abnormal heart development. Additionally, altered brain and/or peripheral serotonin content and/or signaling have been associated with sudden infant death in humans and mice (15–17). Because SSRI inhibit serotonin transporter (SERT; SLC6A4), it modulates serotonin signaling and also affects fetal heart development (18). Nevertheless, the effect of whole-body genetic ablation of serotonin transporter (*Sert*−/−) on fetal outcomes is poorly defined. Moreover, reproductive outcomes and pup survival/death rates for the *Sert−/−* mouse model have been inadequately described.

Mouse is the primary animal model used for biomedical research worldwide (19, 20). The development of mutant mouse models has greatly enhanced the understanding of specific genes in health and disease (20, 21). Yet genetic mouse models can represent a challenge in successfully maintaining mouse colonies due to specific fertility/pregnancy issues or decreased pup survival associated with some genetic lines (22). For example, single gene knockouts are associated with a lethal phenotype in about 25% of mutant mice (21, 23). Aiming to investigate the effects of genetic ablation of *Sert* on reproductive outcomes, we have investigated the reproductive efficiency and occurrence of pregnancy complications in a *Sert−/−* mouse model in light of adverse effects of SSRI treatment during gestation in humans.

## Materials and Methods

### Animals

All procedures in this study were approved by the Animal Care and Use Committee of the College of Agriculture and Life Sciences at the University of Wisconsin-Madison (protocol A005789-R01-02). Animals were maintained in a constant temperature of 25°C, 50–60% humidity, and on a 12/12 h light/dark cycle. All mice received *ad libitum* access to water and mouse chow. Male *Sert−/−* (strain 008355; C57BL/6J background) mice were purchased from Jackson Laboratory (Bar Harbor, ME, United States) and were bred to wild-type females (WT or *Sert*+/+; C57BL/6J; Jackson Laboratory, strain 000664) to produce SERT+/− offspring. Using successive matings, a colony with *Sert*+/− and *Sert−/−* mice was established. Serotonin reuptake in *Sert*+/− mice is similar to WT while it is almost completely ablated in *Sert−/−* (24). Mice were genotyped by PCR amplification using DNA extracted from tail snips (24).

Animals included in this study were either breeding replacements from our colony or were included in another study (only mice receiving no treatment or saline-treatment were used).

Pregnant mice during late pregnancy were observed daily for detection of parturition and evaluation of neonatal mortality. Dams were euthanized due to pregnancy complications on a case-by-case basis. For euthanasia, dams were anesthetized with isoflurane followed by cervical dislocation and exsanguination. Immediately after dam’s euthanasia, the uterus was excised. Using this euthanasia method, viable pups are still alive inside the uterus.

### Animal Breeding

All male breeders were *Sert−/−*. For replacement animals from our colony, two or three virgin females (*Sert−/−* or *Sert*+/−; 8–12 weeks old) were placed in a cage with a male. Starting approximately 15 days after exposure to a male, each female was evaluated to detect pregnancy at least three times per week based on visual observation of abdominal size. Once a female was identified pregnant, she was housed individually. Litters were weaned on postnatal day 21. For the females included from other studies, two or three females (*Sert−/−* or *Sert*+/−; 8–12 weeks old) were placed in a cage with a male overnight and were examined for the presence of vaginal plug in the morning. Detection of vaginal plug confirmed copulation (day post-coitum, DPC0.5).

### Statistical Analysis

All statistical analyses were performed using SAS (version 9.4; SAS Institute Inc., Cary, NC, United States). Data were analyzed with PROC MIXED procedure using one-way ANOVA. Tukey HSH was used for *post hoc* comparisons. Studentized residuals with deviations from assumptions of normality and/or homogeneity of variance were transformed into square root, logarithms, or ranks. For probability of frequency of an event Chi-Square test and Goodness of fit test were used. A probability of ≤0.05 indicated a difference was significant and a probability between >0.05 and ≤0.1 indicated the presence of a tendency for significance. Data are presented as the mean ± standard error of mean (SEM) unless otherwise indicated.

## Results

### Pregnancy Detection

**FIGURE 1:**
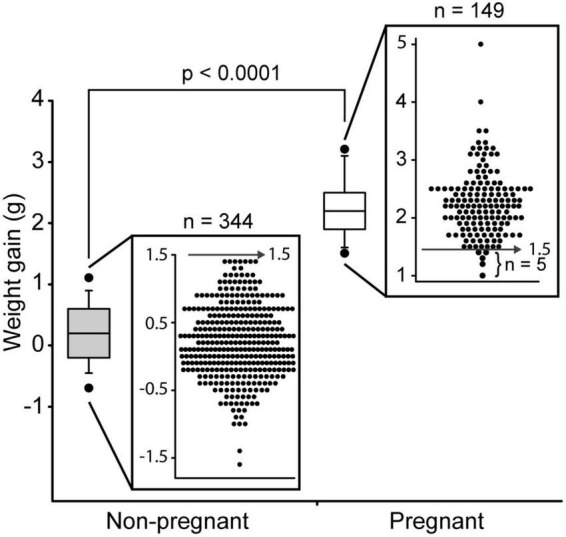
Weight gain between day post-coitum 7.5 and 10.5 in C57Bl/6J mice that became pregnant (*n* = 149) and non-pregnant (*n* = 344). Boxplot of weight gain and weight gain for individual mice is shown. Boxplot depicts median, 5th, 10th, 25th, 75th, 90th, and 95th percentile.

To reliably identify pregnant mice on DPC10.5 (approximately mid gestation), we conducted a survey with previous data from our laboratory. We evaluated the weight gain of C57BL/6J mice between DPC7.5 and DPC10.5. There were 344 mice with a vaginal plug that were found to be not pregnant and 149 mice with a vaginal plug gave birth. The average weight-gain between DPC7.5 and 10.5 was greater in pregnant mice (Figure 1). Using a cutoff value of 1.5 g of weight gain between DPC7.5 and DPC10.5 only 5/149 mice with a vaginal plug were mistakenly identified as non-pregnant, a false-negative rate of 3.4% (96.6% sensitivity). More importantly, no non-pregnant mouse gained more than 1.4 g between DPC7.5 and 10.5, resulting in a false-positive rate of 0% (0/344) so that only pregnant mice are selected based on this method (100% specificity). Establishment of a reliable method for pregnancy diagnosis at DPC10.5 was important for our subsequent evaluations of pregnancy loss after DPC10.5.

### Pregnancy Loss and Preterm Birth

**FIGURE 2:**
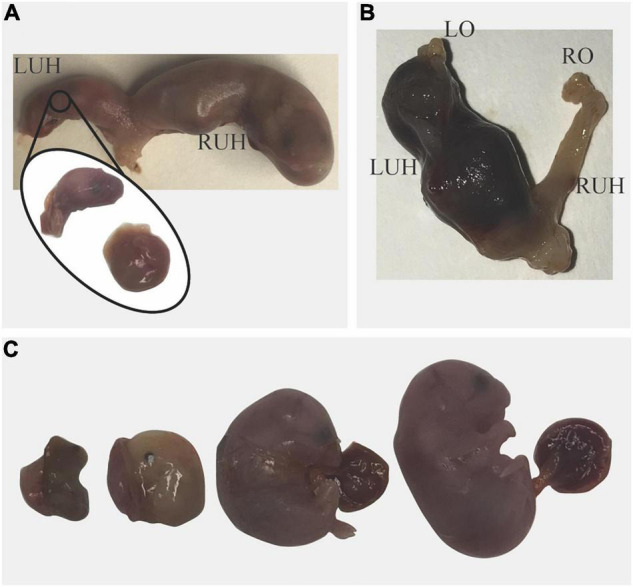
Pregnancy loss in *Sert−/−* mice. **(A)** Blood was observed in the vagina on unknown day of gestation; the dam was euthanized. A viable pup and a dead fetus were found in the right uterine horn (RUH). In the left uterine horn (LUH) only a pale placenta and the head of a fetus were found. **(B)** On day post-coitum 14, blood was observed in the vagina of a dam that lost 1.2 g in 24 h. No fetuses were found in the uterus; in the LUH only blood clots and free blood were found indicating pregnancy loss. **(C)** A pregnant mouse that lost 1.4 g in 24 h was euthanized; All Fetuses were dead and at different stages of resorption.

Pregnancy loss was defined as a female that was deemed pregnant on DPC10.5, based on weight gain, that subsequently did not give birth by DPC19.5 and had no visual indication of still being pregnant, based on abdominal size. As observed in the WT females in our colony analysis, *Sert*+/− females had no pregnancy loss, whereas 17.5% of *Sert−/−* females had pregnancy loss after DPC10.5 (Table 1 and Figure 2).

**TABLE 1:** Pregnancy loss in *Sert*+/− and *Sert−/−* females bred to *Sert−/−* male.

	*Sert*+/−	*Sert−/−*	*p* value
Number of females with a vaginal plug	34	40	
Pregnancy loss after E10.5, *n*	0	7	0.01
Pregnancy loss after E10.5, %	0	17.5	0.01

**FIGURE 3:**
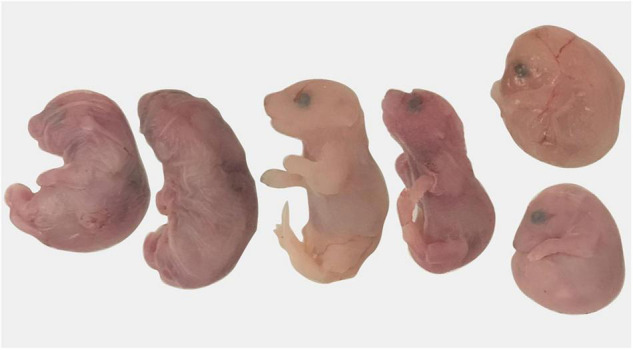
Pregnancy loss in *Sert−/−* mice. A dam gave birth on day post-coitum 18.5; all pups were dead at 9 a.m.

Preterm birth was defined as any female that gave birth prior to DPC19.5. No pregnant *Sert*+/− had premature birth whereas three *Sert−/−* females gave birth on DPC18.5, a day before expected parturition. All pups (6–8 per dam) from these three dams were found dead the morning of DPC18.5 suggesting the pups were born prematurely (Figure 3).

### Prolonged Gestation and Dystocia

**FIGURE 4:**
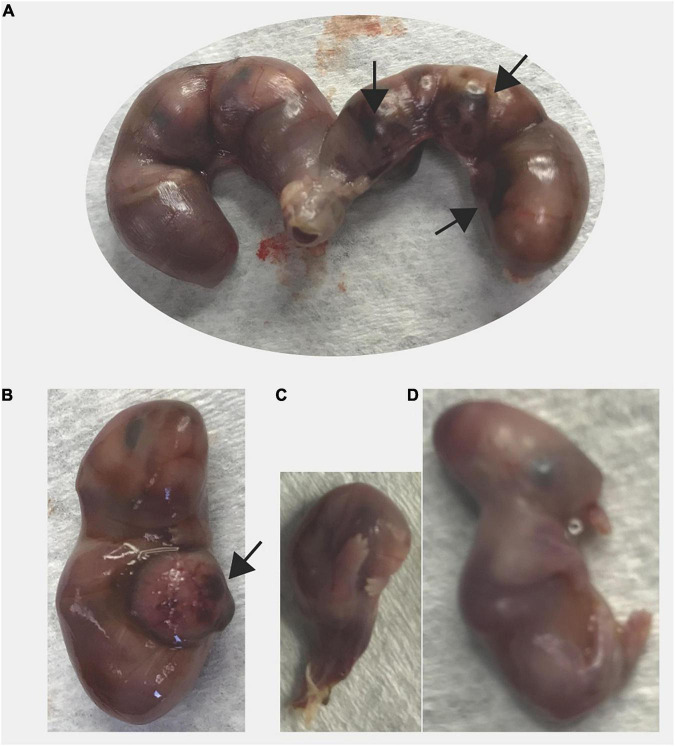
Prolonged gestation in a *Sert−/−* dam. The dam was found with distended abdomen and blood in the vagina on unknown day of gestation. At necropsy of the dam, all fetuses were larger (1.78 g) than average newborn pups (1.3 g) and dead. **(A)** Excised uterus is shown, **(B)** A larger fetus, **(C)** A fetus undergoing resorption, and **(D)** A fetus with abnormal morphology are shown. Black arrow indicates areas of necrosis in the placenta.

We observed two cases of prolonged gestation, both in *Sert−/−* females. One *Sert−/−* female did not give birth by DPC21 and had a very distended abdomen; it was euthanized. Pups were larger than typical newborn pups and 4 out of 9 pups were already dead. As mentioned above, use of our euthanasia method allows viable pups to still be alive inside the uterus. The other dam (*Sert−/−*) was found with blood in the vagina and a distended abdomen; gestational day was unknown. At euthanasia, all six pups were found dead. Pups were very large (1.78 g per pup and 10.68 g overall) which is much larger than the expected weight of six newborn pups (1.2 g per pup and 7.2 g for six pups). Hemorrhage was detected around and within the placenta from most pups (Figure 4). Some pups also had abnormal morphology.

**FIGURE 5:**
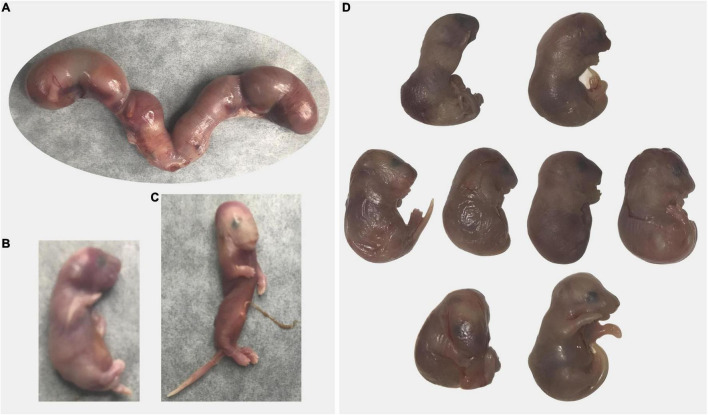
Dystocia in *Sert−/−* mice. **(A–C)** A dam gave birth to three pups that were found dead at 8 a.m.; at 5 p.m. a large abdominal size indicated delivery was incomplete. Excised uterus with four fetuses is shown panel **(A)**. Only one fetus was alive **(B)**. One fetus had abnormal morphology **(C)**. **(D)** A dam was undergoing delivery in the afternoon (one live pup). By the following morning, only one pup has been delivered and was dead and another dead pup was stuck in the birth canal. The dam was euthanized and all retained fetuses were dead **(D)**. Some fetuses presented abnormal morphology.

All mice (*n* = 6) that had dystocia (difficult, abnormal birth) were *Sert−/−* breeders from our colony (Figure 5). Three females had dead pups (one to three) in the cage in the morning and 4–6 h after the dams still appeared to have pups to deliver based on observed large abdominal size. All three dams were euthanized and upon dissection, all retained fetuses were dead (4 or 5 per dam).

One dam gave birth to one pup that was found dead the afternoon of the same day and another dam gave birth to three pups that were found dead the morning after being born. Because of the unusual circumstances (small litter size, dystocia, all pups died), the dams were euthanized. Each of them had retained dead fetuses (one and two, respectively) in the uterus undergoing resorption/mummification.

Lastly, a *Sert−/−* female gave birth to a single pup in the afternoon and had a dead pup stuck in the birth canal the next morning. Upon euthanasia of the dam, seven pups were found dead in the uterus; some pups presented abnormal morphology.

### Litter Size and Pup Survival

To evaluate the effect of maternal genotype on litter size and pup survival before weaning, we followed *Sert−/−* (*n* = 62) and *Sert*+/− (*n* = 55) dams from birth until weaning. Litter size was decreased (88%; *p* = 0.0009) in *Sert−/−* dams compared to *Sert*+/− (Table 2). Additionally, pup death from birth until weaning was greater (234%; *p* < 0.0001) for *Sert−/−* than *Sert*+/− dams resulting in fewer (51.7%; *p* < 0.0001) weaned pups per litter. There was no effect of genotype on pup sex or of pup sex on pup survival.

**TABLE 2:** Litter size and pup survival in *Sert*+/− and *Sert−/−* dams bred to *Sert−/−* males.

	*Sert*+/−	*Sert−/−*	*p* value
Number of dams	55	62	
Pups born, *n*/litter	7.5 ± 0.2	6.6 ± 0.2	0.0009
Pups dead before weaning, %/litter	23.7 ± 4.4	55.5 ± 5.4	<0.0001
Pups dead before weaning, % of pups born	23.4	53.9	<0.0001
Pups weaned, *n*/litter	5.8 ± 0.4	3.0 ± 0.4	<0.0001
Pups weaned, %/litter	76.3 ± 4.4	44.6 ± 5.4	<0.0001
Male pups weaned per litter, %	50.3 ± 3.0	50.2 ± 4.8	0.9
Female pups weaned per litter, %	49.7 ± 3.0	49.8 ± 4.8	0.9

## Discussion

A mouse model with genetic ablation of *Sert* was first reported over two decades ago (24); however, detailed reproductive performance has not yet been adequately reported. Although *Sert−/−* mice are fertile, we observed a series of adverse pregnancy outcomes associated with genetic ablation of *Sert* including: pregnancy loss, congenital malformations, premature birth, dystocia, and perinatal mortality. Although most cases of lethal single gene deletion in mice are associated with prenatal mortality/pregnancy loss (∼50%), perinatal mortality occurs in about 24% of genetic models carrying lethal mutations (22). Interestingly, *Sert−/−* mice experienced both pregnancy loss and perinatal mortality. Although we do not show a direct effect of SSRI on the occurrence of adverse pregnancy outcomes in the present manuscript, we demonstrate a role for adequate serotonin transporter function for successful pregnancy outcomes and neonatal health. Of particular importance, our findings for the *Sert−/−* mouse model coincide with the reported effects for SSRI treatment during gestation on pregnancy outcomes in humans. Although not compared in this study, reproductive outcomes in *Sert*+/− and WT dams are similar (Domingues and Hernandez, unpublished results) since serotonin reuptake in *Sert*+/− mice is similar to WT mice (24).

Platelet SERT allows entry of serotonin into platelets, thereby controlling free circulating (plasma) concentrations of serotonin. Therefore, lack of SERT (*Sert−/−*) or pharmacological inhibition of SERT (SSRI treatment) results in increased plasma concentrations of serotonin (9, 25, 26). Previous studies using 5-HTP (serotonin precursor) to elevate circulating serotonin have observed reduced uterine/placental blood perfusion and increased pregnancy loss (27, 28). This is consistent with the decrease in uterine artery blood flow that has been observed during SSRI treatment (29). In addition, the decreased uterine/placental perfusion caused by increased serotonin compromises placental function and results in placental pathology (30). Indeed, placenta collected from *Sert−/−* dams had abnormal hemorrhage, and increased necrosis and fibrosis (25). More recently, abnormal placental morphology and placental gene expression were reported in the placenta of *Sert*−/− conceptus (31). Similarly, placenta collected from women undergoing SSRI treatment during gestation had increased vascular lesions including hemorrhage and fetal thrombo-occlusive disease (32). Pathology and malperfusion of the placenta result in placental insufficiency, the main cause of fetal growth restriction, a common cause of perinatal mortality and preterm birth (33). Thus, the pregnancy loss and preterm birth observed in the *Sert−/−* dams in the present study are likely to be due to placental malperfusion and insufficiency triggered by the elevated plasma serotonin in mice lacking SERT. Moreover, pregnancy loss in the present study occurred after the fetus became completely dependent on the placenta (after DPC10.5 in mice) further suggesting a role of placenta function on pregnancy loss (34).

A perinatal lethal phenotype occurs in about 24% of mutant mice (22). The most common causes for neonatal mortality associated with a lethal phenotype in mice are cardiorespiratory, neuromuscular, skeletal, craniofacial, and metabolic defects (22, 35). Additionally, maternal behavior may affect pup survival. In the present study perinatal mortality, either due to dystocia or neonatal death, was clearly increased in *Sert−/−*. Noteworthy, dystocia and neonatal death are increased in mice treated with either fluoxetine or sertraline (two of the most popular SSRI) during gestation (36). Perhaps the lack of reports of dystocia in women and neonatal death in infants exposed to SSRI *in utero* are due to more prompt medical assistance and interventions, in contrast to laboratory animals. However, other labor-associated complications such as post-partum hemorrhage are increased in women taking SSRI during gestation (37, 38) and neonatal morbidity is increased in babies exposed to SSRI *in utero* resulting in increased neonatal admission into NICU (1, 2, 4).

Altered serotonin signaling in mutant mouse models [serotonin receptors 2B (12), 3A (14) and *Sert* (25, 39, 40) knockouts] or pharmacological manipulation of SERT (SSRI treatment) during pregnancy in WT mice (18) has been associated with abnormal heart development resulting in pre- and perinatal death. Additionally, a role for SERT on cardiac pathology has been described (25, 39–41). Therefore, not surprisingly, some SSRI cause major cardiac malformations in humans (3). Interestingly, *Sert−/−* mice (25, 31) and women taking SSRI during gestation (32) have several placental pathologies which have also been associated with altered embryonic/fetal cardiovascular development (34). Furthermore, sudden infant death syndrome has been associated with abnormal serotonin signaling such as: increased serum concentrations of serotonin (17), multiple brain serotonergic abnormalities (42, 43), and polymorphisms in the *Sert* promoter region (39). Therefore, although the cause of neonatal mortality was not investigated in the present study, it reproduced the sudden infant death syndrome phenotype observed in humans and other mouse models (39) and is likely to be associated with similar cardiac pathology as observed in those conditions. However, fetal/neonatal morphological defects were also observed in the present study, and these might underlie at least some of the cases of fetal/neonatal mortality. Further studies are needed to investigate the role of altered SERT function, either by genetic ablation of *Sert* in mice or pharmacological inhibition of SERT, on cardiac development and its role in perinatal morbidity/mortality and sudden infant death syndrome.

Responsible biomedical research involving animals includes reducing and refining animal use in research while still acquiring adequate and useful data. In studies of pregnancy in mice, an accurate method for early pregnancy diagnosis can be particularly useful. Our method for pregnancy diagnosis in mice provided 100% specificity and a sensitivity greater than 96% for identifying pregnant dams by mid gestation (DPC10.5). Implementing this method was invaluable for identifying pregnancy loss after DPC10.5 in *Sert−/−* females, previously overlooked and/or unreported information that may shed light on the role of serotonin in pregnancy maintenance. This simple and accurate pregnancy diagnosis method may be useful for other studies of pregnancy loss in mice.

In conclusion, the similarities between the occurrence of adverse pregnancy outcomes in *Sert−/−* mouse model and women and mice undergoing SSRI treatment during gestation suggest a critical role for SERT in pregnancy maintenance, fetal development, and neonatal health. Hence, *Sert−/−* mice might be a useful model to comprehensively understand how altered serotonin signaling leads to pregnancy complications and neonatal morbidity/mortality in SSRI-exposed mammals. Additionally, it suggests that the adverse pregnancy outcomes in women undergoing SSRI treatment are due to altered SERT function due to SSRI and are independent of the underlying depression. This is a critical finding given the number of women that are currently prescribed SSRIs during pregnancy (5) and provides the framework for future research in this area.

## Data Availability Statement

The original contributions presented in the study are included in the article, further inquiries can be directed to the corresponding author.

## Ethics Statement

The animal study was reviewed and approved by Animal Care and Use Committee of the College of Agriculture and Life Sciences at the University of Wisconsin-Madison (protocol A005789-R01-02).

## Author Contributions

RD: conceptualization, methodology, validation, formal analysis, investigation, data curation, writing—original draft, writing—review and editing, visualization, and project administration. MW and LH: conceptualization, methodology, resources, writing—review and editing, visualization, supervision, and funding acquisition. All authors contributed to the article and approved the submitted version.

## Conflict of Interest

The authors declare that the research was conducted in the absence of any commercial or financial relationships that could be construed as a potential conflict of interest.

## Publisher’s Note

All claims expressed in this article are solely those of the authors and do not necessarily represent those of their affiliated organizations, or those of the publisher, the editors and the reviewers. Any product that may be evaluated in this article, or claim that may be made by its manufacturer, is not guaranteed or endorsed by the publisher.
